# Functional orthogonality of parvoviral phospholipase A_2_ domains in adeno-associated virus transduction

**DOI:** 10.1128/jvi.00799-25

**Published:** 2025-08-12

**Authors:** Joshua A. Hull, Robert M. Fusco, Jeffery Tan, Mark A. Ochoa, Aaron Hall, Xinlong Wan, Ezra Loeb, Aravind Asokan

**Affiliations:** 1 Department of Surgery, Duke University School of Medicine 12277 , Durham, North Carolina, USA; 2 Department of Biomedical Engineering, Duke University Pratt School of Engineering 101571 , Durham, North Carolina, USA; 3 Department of Molecular Genetics and Microbiology, Duke University School of Medicine 12277 , Durham, North Carolina, USA; Cornell University Baker Institute for Animal Health, Ithaca, New York, USA

**Keywords:** intracellular trafficking, parvovirus, phospholipase A2, adeno-associated virus, transduction, infectious pathway, nuclear import/export, endosomal escape

## Abstract

Dependoparvoviruses, which belong to the family *Parvoviridae,* are being developed as viral vectors for gene transfer. Notably, different adeno-associated viral (AAV) serotype capsids have been utilized to generate pseudotyped recombinant vectors. While capsid surface regions mediate host cell interactions, buried structural domains have been implicated in parvoviral infectivity and post-entry trafficking. In this regard, the functional diversity of highly conserved group XIII phospholipase A_2_ domains (PLA_2_) located within the N-terminal capsid domain of different parvoviruses is of particular interest. Here, we developed a massively parallel screen to evaluate a diverse panel of rationally engineered and naturally derived parvoviral PLA_2_ domains incorporated within the human isolate, AAV9. *In vitro* infectious cycling of chimeric virions revealed a functional bias toward parvoviral PLA_2_ domains of mammalian and avian origin and decreased preference for PLA_2_ domains of insect, ungulate, or metagenomic origin. Notably, wild-type chimeric AAV9 virions carrying avian dependoparvovirus PLA_2_ domains demonstrate increased replication over other chimeras. The best-performing recombinant avian/human origin chimera (UNY47950.1/AAV9) shows improved transduction with both single-stranded and self-complementary vector genomes. This observation is accompanied by improved cytoplasmic uptake and nuclear entry of chimeric virions compared to parental AAV9, as evidenced by subcellular fractionation and confocal microscopy. Overall, this study highlights the functional orthogonality of distinct parvoviral PLA_2_ domains incorporated into AAV capsids. These chimeric virions present an opportunity to gain deeper insight into the infectious biology of parvoviruses and potentially enable new approaches to improve post-entry trafficking of AAV vectors for gene transfer applications.

**IMPORTANCE:**

This study explores the functional overlap of phospholipase domains located within the capsid lumen across the parvovirus family. The findings provide insights into parvovirus-host interactions across different genera within the context of this highly conserved capsid region and underscore its essential role in viral trafficking to the nucleus. Furthermore, incorporation of orthogonal phospholipase domains derived from diverse parvoviral family members may expand the recombinant vector toolkit of adeno-associated viruses for gene transfer applications.

## INTRODUCTION

*Parvoviridae* are a family of non-enveloped, icosahedral single-stranded DNA packaging viruses (1). Pathogenic isolates are found to infect vertebrates and non-vertebrate eukaryotes, while non-pathogenic isolates are best characterized by vertebrate-infecting *Dependoparvoviruses* (1). The latter, which include adeno-associated viruses (AAVs), have been extensively studied and developed as recombinant vectors for gene therapy applications, most notably in the treatment of monogenic disorders (2, 3). Structurally, the parvoviral capsid is a non-enveloped T = 1 icosahedron made of minor and major viral capsid proteins (VPs) that are generated by alternative splicing and leaky start site recognition, resulting in a shared C-terminal region and minor VP having additional N-terminal extensions (1, 4–7). In the case of the AAV *Cap* gene, this process is primarily driven by the P40 promoter located within the *Rep* gene that yields a ratio of 1:1:10 for VP1, VP2, and VP3 capsid subunits (6). VP3 forms the major capsid subunit, while VP1/2 share an N-terminal domain, and VP1 has a unique N-terminal domain (VP1u) (1, 4). The VP3 subunit is the core structural component of the icosahedral capsid, which is characterized by an eight-stranded *β*-barrel jelly roll motif, typically with an accessory *β*-strand A (5). Surface structural features on the capsid formed by interdigitation of VP3 variable loop regions are responsible for cellular glycan and protein receptor binding interactions and are the most common target of serological immune recognition and neutralization (5, 8–13). Two additional proteins, assembly activating protein (AAP) and the membrane-associated accessory protein (MAAP), are also expressed with roles in viral particle assembly and cellular egress, respectively (14).

The minor capsid protein domains, such as VP1u, also play a critical role in parvoviral infectivity but remain comparatively underexplored. For instance, the VP1u region is required for multiple processes, including post-entry receptor binding, nuclear localization, endo-lysosomal escape, and preventing epigenome silencing (15–24). It has been demonstrated for most parvoviruses (except *Erythroparvovirus*) that VP1u is hidden within the capsid until externalization is triggered during viral transduction, following cell binding and entry (13, 19, 20, 25, 26). A key functional domain of VP1u is a group XIII phospholipase A_2_ enzyme (PLA_2_), a conserved enzymatic motif essential for endo-lysosomal escape (16, 19, 21–23, 27). Unlike other PLA_2_ superfamily members, group XIII domains lack stabilizing disulfide bonds, a unique feature hypothesized to facilitate its passage through the fivefold pore (~8 Å) (13, 25, 27–29). Group XIII and group III (bee venom) PLA_2_ are closely related folds, and the active site residues, calcium binding loop, and location of the lost disulfides can be inferred (23, 30). Furthermore, all parvoviral PLA_2_ domains have unique rates of reaction toward cleaving different phospholipid substrates, including unique pH optima (19, 22). In addition, certain PLA_2_ of both host and venom origin bind to and activate host receptors (notably PLA_2_R) in addition to their enzymatic activity (31, 32). Despite being essential for infection, the structural and evolutionary constraints governing PLA_2_ function and cross-species compatibility are poorly defined.

To assess the functional orthogonality and domain determinants of parvoviral PLA_2_ domains, we constructed a comprehensive library of 779 natural parvoviral PLA_2_ domains spanning multiple genera, along with 199 synthetic chimeras and 16 engineered disulfide-stabilized variants, introduced into AAV as a template. Using high-throughput library screening, structural modeling, and functional assays, we aimed to identify determinants of PLA_2_ compatibility and potential avenues for AAV transduction enhancement. Our study provides new insights into the evolutionary and structural constraints of PLA_2_, while identifying avian-derived PLA_2_ sequences that improve AAV transduction. These findings provide mechanistic insights into PLA_2_ evolution and structure-function relationships while establishing a framework for enhancing intracellular trafficking in AAV vectors to improve gene therapy applications.

## MATERIALS AND METHODS

### PLA_2_ identification and library design

**Fig 1:**
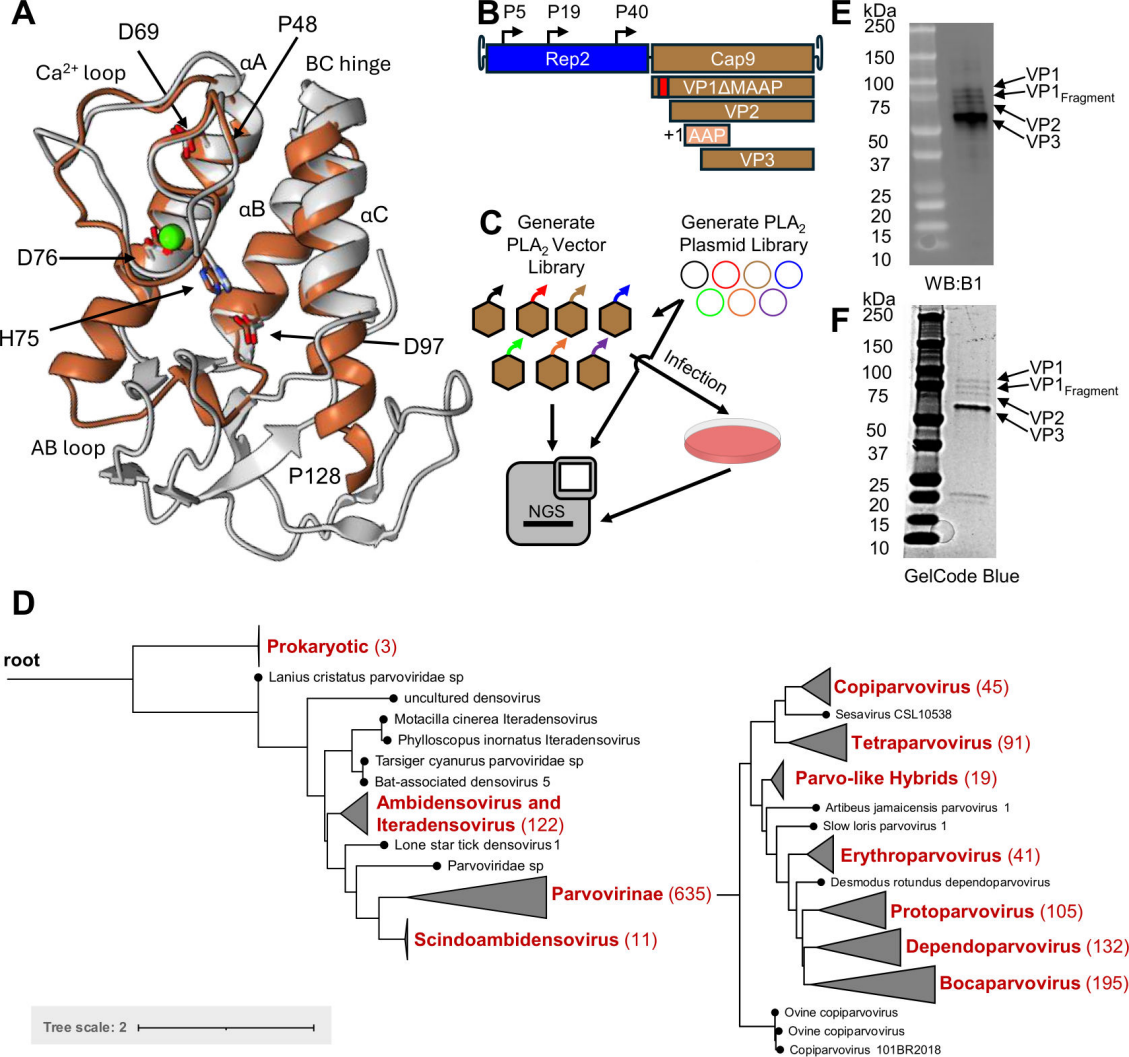
Chimeric AAV-parvoviral PLA_2_ library construction and generation. (**A**) The structure of the core PLA_2_ domain of AAV9 (brown) and bee venom PLA_2_ (type III prototype, pdbid: 1POC, gray) superimposed, highlighting core motifs such as the catalytic dyad and conserved Ca^2+^ binding Asp, and the first and last amino acids in the PLA_2_ swapped domain (P48 and P128). (**B**) Representation of the wtAAV2/9 cassette used in evolution with a deletion of MAAP9. The region swapped is highlighted in red. (**C**) Graphical representation of workflow for *in vitro* cycling. The swap library was first cloned into a vector plasmid, then used to produce vector (P0), and subsequently cycled *in vitro* on HEK293 cells to generate P1 and P2. All steps were sequenced with next-generation sequencing (NGS) to confirm library distribution at each step. (**D**) Phylogenetic tree of the tested natural parvoviral PLA_2_ domains. Tree root is shown on the left, with large clades collapsed and the *Parvovirinae* clade expanded on the right. Collapsed nodes are colored in red with the number within the collapsed node shown in parenthesis. A tree scale in amino acid substitutions/distance corresponding to both is shown at the bottom left. (**E**) Western blot of the PLA_2_ library preparation. (**F**) SDS-PAGE gel of PLA_2_ library preparation.

We conducted a comprehensive literature review to identify conserved parvoviral PLA_2_ domains. Using the conserved sequence motif DxxxxxHD characteristic of PLA_2_ groups III and XIII. Furthermore, with AlphaFold2, we identified residues P48–P128 in AAV9 as the core fold of the PLA_2_ domain (33). This analysis yielded 779 parvoviral PLA_2_ domains with regions homologous to AAV9’s PLA_2_ domain (Fig. 1A).

To explore structural constraints and potential functional enhancements, we designed synthetic variants incorporating predicted disulfide bonds. Disulfide positions were inferred from group III PLA_2_ structural alignments and introduced at the AAV9-equivalent residues defined in Table 1. Additionally, novel PLA_2_ were generated using a generative Markov matrix-guided approach to maximize the library diversity (34). Briefly, a set of predicted protein structures was generated by AlphaFold2 (33), filtered by structural similarity to a reference model (AAV9 PLA_2_ domain), based on Cα root-mean-squared deviation (RMSD). Structures with RMSD > 2.0 Å were excluded, leaving 484 PLA_2_ domains. From the remaining models, residue-residue contacts were identified using a 5.0 Å cutoff, and contact profiles were generated for each sequence. A contact-based profile model was constructed to guide sequence design, incorporating tolerated amino acid substitutions from the BLOSUM90 matrix (those substitutions with a log likelihood > 0). Synthetic sequences were stochastically generated by sampling from this model, ensuring compatibility with observed contact patterns.

**TABLE 1:** PLA_2_ domain disulfide substitutions within the AAV9 VP1 subunit^*a*^

Disulfide no.	Substitution 1	Substitution 2
C1	L53C	A72C
C2	A71C	R103C
C3	A78C	A96C
C4	K92C	L126C

^*a*^ Predefined disulfide substitution pairs used within the study.

To assist selection and next-generation sequencing (NGS), a codon substitution protocol was used to ensure that (i) all variants possessed a silent barcode in the forward direction for quantification and (ii) no additional ORFs existed in the context to prevent chimeric MAAP expression.

Wild-type parvoviral libraries were cloned by first introducing the chimeric capsid genes containing parvoviral PLA_2_ domain encoding sequences embedded within the AAV9 *Cap* gene into a Rep2/Cap9 shuttle vector (35). The pooled chimeric capsid clones were then further subcloned into a plasmid containing AAV2 inverted terminal repeats (35) for propagation (Fig. 1B). Library production was done by standard transfection method previously published for wild-type vector production (35). Briefly, a single 15 cm plate of HEK293 cells grown in DMEM (ThermoFisher Cat# 11995-065) supplemented with 10% (vol/vol) FBS was cultured in 5% CO_2_ at 37°C. At 80% confluence, this plate was transfected with 6 µg of the library plasmid, 10 µg of pHelper (XX680), which contains essential adenoviral genes for AAV vector production, and 56 µg of PEI (PolySciences Cat# 49553-93-7). The cell pellet and media were harvested 6 days later. The media were incubated with 10% (wt/vol) PEG8000 (Sigma-Aldrich Cat# P2139-2KG), then virus was precipitated by spinning at 3,500 × *g* for 45 min. Cells were lysed by 3× freeze-thaw on dry ice. Cells and media were pooled, treated with DNase I, and adjusted to 1 M NaCl. Next, the mixture was loaded onto an iodixanol step gradient with 17% iodixanol in 1× PBS, supplemented with 0.5 M NaCl; 25% iodixanol in 1× PBS; 40% iodixanol in 1× PBS; and 60% iodixanol. The gradient was spun in a wX+ Ultra Series centrifuge (Thermo Scientific) at 30,000 rpm in a Sorvall Surespin 630 (Thermo Scientific) swinging bucket rotor for 16 h. The 40% fraction was extracted by the bottom puncture method and buffer exchanged using a 100 kDa concentrator (Thermo Scientific Cat# 88524).

### Structural modeling

Structural modeling was done with AlphaFold2 at the initiation of the study to infer the positions of disulfides in the structure of AAV9 (33). Comparing the fold topology of different PLA_2_ groups, AlphaFold3 was used (36). Structural images were generated in ChimeraX (37).

### Phylogeny

A phylogenetic tree was generated with the interactive tree of life webserver (38). Proteins were aligned by the DxxxxxHD motif, with no insertions/deletions allowed.

### AAV genome quantification

AAV genome quantification was done according to previously published protocols against the ITRs (35). Briefly, 2 µL of virus was added to 198 µL of DNase buffer (10 mM Tris-HCl, 10 mM MgCl_2_, 2 mM CaCl_2_, and 0.1 mg/mL DNase I), then incubated for 1 h at 37°C. DNase I was inactivated first by the addition of 5 µL of 0.5 M EDTA, pH 8.0, then by incubation at 55°C for 15 min for irreversible denaturation. Next, 2 µL of sample was added to 198 µL of molecular biology-grade water. Finally, 2 µL of each sample was combined with 5 µL SYBR Green qPCR Master Mix (Roche Cat# 04707516001), 2.5 µL H_2_O, and 0.5 µL primer mix (10 µM each forward and reverse primer) and quantitated against a known reference standard. For qPCR detection, the sample was denatured for 10 min at 95°C, then subjected to a three-step protocol with the following steps repeated: 45 cycles at (i) 95°C for 10 s, (ii) 62°C for 10 s, and (iii) 72°C for 10 s. The primers (ITR-F 5′-AACATGCTACGCAGAGAGGGAGTGG-3′ and ITR-R 5′-CATGAGACAAGGAACCCCTAGTGATGGAG-3′) were used for detection.

### *In vitro* infectious cycling

The day before transduction, a plate of adherent HEK293 cells was transfected with XX680 at 80% confluence. Sixteen hours later, the media were replaced, and the cells were given 2 h to recover. Cells were then transduced at a multiplicity of infection (MOI) of 10^5^ vg/cell. The supernatant was harvested 5 days later for vector quantification, NGS analysis, and subsequent viral passaging. The first cycle from the parental plasmid produced virus (P0) generates the viral progeny 1 (P1), while the second time generates the viral progeny 2 (P2). This cycling method allowed for the deselection of viral variants that were unable to produce progeny outside of the context of cross-packaging. At the transduced MOI, it is expected that most transduced cells will only have a small number of successful viral genomes to generate relatively pure progeny.

### Next-generation sequencing and analysis

For NGS, the virus was treated with DNase I as previously described (35) to remove contaminant DNA, while the parental plasmid was not DNase I treated. The genomic pool was amplified by PCR using primers targeting the swapped PLA_2_ domain with adapters for the minimum required cycles (PLA_2_fwdEZ 5′-ACACTCTTTCCCTACACGACGCTCTTCCGATCTACGCTCGAGGTCTTGTGCTT-3′ and PLA_2_revEZ 5′-GACTGGAGTTCAGACGTGTGCTCTTCCGATCTGCTTCCTCAACCAGACCAAG-3′) and sequenced using Azenta amplicon EZ service as previously published (35). The sequences were then compared to the known exact nucleotide reading frame with a python script, and non-matching sequences were discarded. A schematic of *in vitro* cycling and sequencing is shown in Fig. 1C.

### Embedding and clustering of PLA_2_ sequence information

To visualize and assess the sequence diversity, along with species/host interacting space within the PLA_2_ domains, we applied a deep-learning language model-based embedding approach. The primary sequence of the natural PLA_2_ domains was embedded as a numerical protein representation with ESM2 model t6_8M_UR50D taking the last embedding layer (39). This embedding layer is a high-dimensional numerical representation, which has been demonstrated to capture biochemical, evolutionary, and structural features (39). To reduce the dimensionality for interpretation, this embedding layer was visualized by splitting the embedded layer into two components by t-distributed stochastic neighbor embedding (t-SNE) using scikit-learn (40). The resulting two-dimensional representation was plotted with matplotlib (41).

We subsequently labeled the data with the known host of isolation and the genera of virus. This was used to classify PLA_2_ domains in a supervised manner for the interpretation of the host/genus interacting space.

### Production of recombinant AAV vectors

Virus was produced as standard Rep2/Cap9 plasmids for triple transfection vector production, as has been previously described (42). Briefly, suspension-adapted HEK293 cells grown at a cell density of 2 × 10^6^ cells/mL in FreeStyle F17 media (ThermoFisher Cat# A13835-01) were triple transfected with 0.6 µg/mL of pHelper (XX680), 0.5 µg/mL of pRep2/Cap, 0.3 µg/mL of ITR reporter, and 4.9 µg/mL of PEI (PolySciences Cat# 49553-93-7). The Cap9 ORF consisted of a PLA_2_ encoded by the wild-type nucleotide sequences or the minimal number of mutations in the case of disulfides. Vector was purified after 6 days of transfection by iodixanol gradient ultracentrifugation and buffer exchange as previously described (35, 42). Vector genome titers were obtained using qPCR as previously described (35).

### Viral mRNA quantitation

Standard triple transfection was done in HEK293 cells, which were harvested on day 3. The cells were lysed with SideStep reagent (Agilent Cat# 400900-21). The RNA input quantity was determined using RNA Qubit (Thermo Fisher Cat# Q32852). Detection of mRNA was carried out using the QIAcity OneStep Advanced Probe Kit (Qiagen Cat# 250131) and custom primers and probes (Table 2). The cell lysates were diluted and added to a 96-well format 8.5k plate (Qiagen Cat# 250011). For analysis, P5/19 positive wells were removed to prevent any false positives of the P40Minor products arising from these promoters. The dilution factor was used to back-calculate the number of transcript copies per μL loaded, and the resuspension volume and total RNA yield were used for normalization calculation.

**TABLE 2:** Primers and probes used for the quantification of P40 major/minor transcripts^*a*^

	P5/19	P40Major	P40Minor
Forward primer	GCGGCTTCCGTCTTTCTGGGATG	GACGCGGAAGCTTCGATCAAC	GACGCGGAAGCTTCGATCAAC
Reverse primer	CAGTGCAAGCGTCTGGCACCTTTC	CGGAAGCACAAGACCTCGAGC	CGGAAGCACAAGACCTCGAGC
Probe	5HEX/CCGGTGGGC/ZEN/AAAGGATCAC	5ATTO550N/CTTAGTGAAGG AATTCGCGAGTGGTG/3IAbRQSp	5Cy5/GGTATGGCT/TAO/GC CGATGGTTATCTTCC/3IAbRQSp

^*a*^ Primer/probe sets used in the mRNA quantification experiments.

### Mass photometry

Mass photometry was carried out using the Refeyn SamuxMP system for the estimation of full and empty capsids. For focus calibration, 10–18 μL of PBS was added, and then focus was determined. After focusing, the volume was brought to 20 µL with virus, and a 1-min video was recorded and analyzed on AquireMP and DiscoverMP software, respectively (Refeyn). For mass calibration, AAV9 empty capsids produced in a Rep-lacking system were used as the empty control (3,800 kDa), while wild-type Rep2/Cap9 virus was used as the full standard (5,250 kDa). After calibration, full mass was defined by the following formula:


$$Full\;mass=\frac{[(genome\;length\;in\;bp)\times 0.61596\;kDa]+0.03604\;kDa}{2}+3800\;kDa$$

Full particles were quantitated within ±250 kDa of full-particle peak.

### Luciferase expression assay

HEK293 cells were seeded at 2 × 10^4^ cells/well in 180 µL/well in a white 96-well plate 2 h before experiments. The virus was added to a final volume of 20 µL in PBS to transduce cells at the given multiplicity of infection per experiment in triplicate. After 48 h, cells were lysed with passive lysis buffer (Progen Cat# E194A), luciferase reagent was added (Progen Cat# E1483), and then read on Thermo Scientific Varioskan LUX.

### Flow cytometry assay

HEK293 cells were seeded at 1 × 10^5^ cells/well in 900 µL/well in a 24-well plate on the morning of the experiment and allowed to recover for 2 h. The virus was added to a final volume of 100 µL in PBS to transduce cells at the given multiplicities of infection. After 48 h, cells were dissociated from the plate with 0.05% trypsin, then resuspended in DMEM supplemented with 10% FBS. Cells were pelleted at 500 × *g* for 5 min, DMEM was aspirated, and resuspended in PBS. Cells were filtered through a 0.4 µm cell strainer. Flow analysis was performed on the BD LRS Fortessa X20 or Sony MA900 cell analyzer. Flow cytometry data were generated by first gating on live cells, followed by doublet discrimination (FSC-A by FSC-H), and subsequently on GFP (FITC+). Experiments were performed in triplicate. Data were analyzed using FlowJo version 10.8.1 (BD Biosciences).

### Cellular and subcellular quantitation assays

Cell binding assays were done with HEK293 cells in a 96-well format with 1 × 10^5^ cells/well seeded 2 h before experiments, then put on ice for 5 min. After incubation, the vector was added at an MOI of 10^5^. Cells were moved into a cold room at 4°C onto a rocker and incubated for an hour. After 1 h, the media were aspirated, and the cells were resuspended in 50 µL of SideStep reagent (Agilent Cat# 400900-21). Lysates were frozen at −20°C until processed.

For nuclear and cytoplasmic localization, HEK293 cells were seeded in a 24-well plate at 1 × 10^5^ cells/well and allowed to recover for 2 h. Luciferase vector was added at 10^5^ MOI and harvested 16 h later. Nuclear and Cytoplasm Kit from Thermo Scientific (Cat# 78835) was used with modifications. Lysis of the cytoplasm with buffers (CER) from the kit was done as per protocol, but the lysis of the nuclear envelope with buffers (NER I and NER II) was omitted due to incompatibility with PCR methods, and SideStep reagent was used instead (Agilent Cat# 400900-21). Lysates were frozen at −20°C until processed.

For both experiments, the fractions were diluted as appropriate and added to a 96-well 8.5k plate (Qiagen Cat# 250011). A Qiagen QIAcity One was used with the dPCR CGT Assay ITR2/5 (HEX) kit (Qiagen Cat# 250231). Total vector genome recovered was back-calculated from the determined concentration, dilution factor, and starting resuspension volume.

### Immunofluorescence and confocal microscopy

HEK293 cells at 2 × 10^4^/well were seeded onto a gamma-irradiated 35 mm glass bottom poly-D-lysine-coated dish (Mattek Cat# P35GC-1.5-14-C) and transduced with a luciferase vector at a multiplicity of infection of 2 × 10^5^. Cells were harvested 16 h post-transduction.

Cells were fixed with 4% paraformaldehyde in PBS for 15 min at room temperature, followed by permeabilization with 0.5% Tween 20 in PBS for 5 min. Non-specific binding was blocked with 1% bovine serum albumin in PBS with 0.5% Tween 20 for 30 min. Cells were then incubated with primary antibody (ADK9 hybridoma supernatant targeting AAV capsid protein, diluted 1:1,000 in blocking buffer) overnight at 4°C. After washing with PBS, secondary anti-mouse antibody conjugated to GFP was applied for 1 h at room temperature in the dark. ADK9 was a gift from the lab of Mavis Agbandje-McKenna and Robert McKenna.

Following additional PBS washes, nuclei were counterstained with DAPI (0.1 µg/mL for 5 min), and coverslips were mounted using mounting medium. Imaging was performed using a Zeiss 880 inverted confocal microscope with Airyscan. After acquisition, channels were separated, and the blue channel (DAPI) was used to generate a nuclear mask. This mask was applied to the GFP channel to identify GFP signal within nuclear regions. GFP fluorescence intensity was quantified using the corrected total cell fluorescence formula (Corrected total fluorescence intensity = integrated density – (area of ROI × mean background fluorescence). Image processing and quantification were performed using Fiji (ImageJ).

### Statistical analysis

Statistics were calculated in GraphPad Prism version 10.1.2. When a single comparison was made, a Student’s *t*-test was used. For multiple comparisons, ANOVA was used, assuming paired measurements when appropriate (e.g., experiments done on multiple days with reference control), and otherwise using unpaired measurements. Outliers were identified and removed using the ROUT method with *Q* = 1%.

## RESULTS

### High-throughput selection of orthogonal parvoviral PLA_2_ domains

To systematically evaluate PLA_2_ compatibility, a wild-type library was synthesized containing 1,000 unique PLA_2_ domains of parvoviral origin. The library also included synthetic chimeras that were structurally modified to contain disulfide bridges, as well as variants carrying inactivating mutations for use as negative control. Domains of natural parvoviral origin span broad evolutionary diversity (Fig. 1D). To confirm protein expression from the library, we performed western blot (Fig. 1E) and SDS-PAGE (Fig. 1F). The library candidates expressed VP1, VP2, and VP3, with some additional products observed between VP1 and VP2 bands, presumed to be truncated PLA_2_ domains. These defective sequences may arise from multiple sources, including aberrant splicing, proteasomal degradation, or VP initiation from leaky start codons.

**Fig 2:**
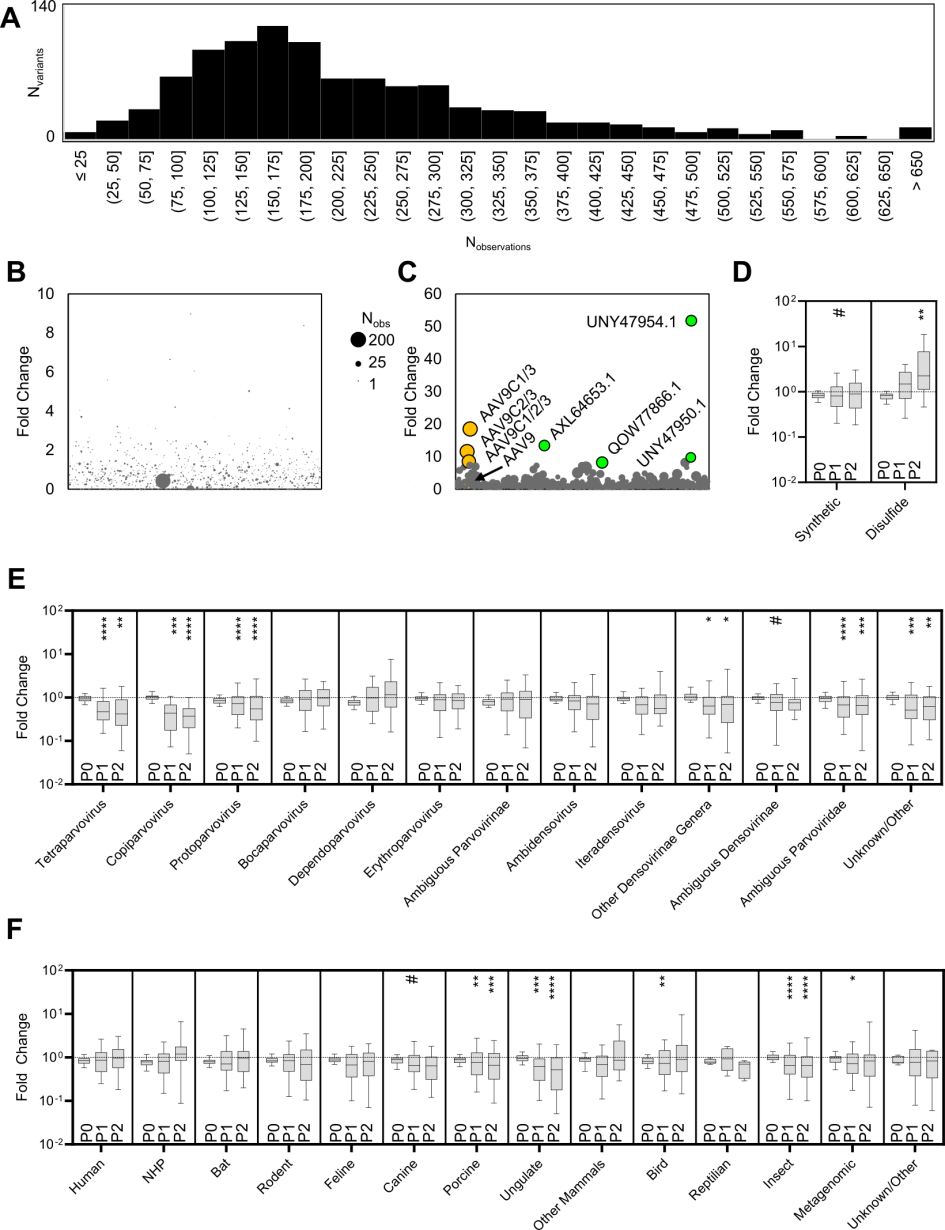
*In vitro* library cycling yields functionally compatible PLA_2_ domains from different parvoviral isolates, genera, and host sources. (**A**) Starting distribution of the PLA_2_ wild-type variants produced by transient transfection at P0 used in *in vitro* cycling. After viral infection and expansion *in vitro,* the variants were sequenced, and the change in their distribution is plotted as bubble plots of (**B**) P1 and (**C**) P2. Within the bubble plots, variants of interest are colored yellow (disulfide containing), green (natural isolate), or brown (wtAAV9) and labeled. Fold change over multiple passages (P0, P1, and P2) for (**D**) synthetic and AAV9 disulfide-containing variants, (**E**) viral genera of origin, or (**F**) host of origin plotted as a box and whiskers plot with whiskers from the 5th to the 95th percentile for variants that were detected in selection cycles. Each group shown had a minimum of five variants. One-way ANOVA against P0: #*P* < 0.1; *P < 0.05; **P < 0.01; ***P < 0.001; and ****P < 0.0001.

NGS was used to assess variant representation in the wild-type parvoviral library. We confirmed the presence of all synthesized variants in the plasmid and initial vector library (P0) in our NGS reads by comparing to the known nucleotide sequences (Table S1). The initial representation followed a unimodal distribution with a momental rightward skew of +31.6 toward higher represented variants (Fig. 2A). Prior studies have reported that for AAV libraries, cross-packaging (wherein an AAV capsid packages an unassociated AAV genome, not encoding the corresponding reading frame) is significant in the first produced library from plasmid transfection due to the high number of plasmid copies used per cell (43). To mitigate this, we opted for iterative selection rather than reducing the plasmid input. Given that initial transduction efficiency is expected to be low, we hypothesized that *in vitro* infectious cycling should provide sufficient stringency and selective pressure required for natural selection of dominant clones. Consistent with this expectation, the first round (P1) of infectious viral cycling showed modest change in variant distribution (Fig. 2B) compared to the second round (P2) of infectious cycling (Fig. 2C).

Within the second round of infectious cycling, the top enriched variants included those harboring synthetic disulfide bonds and natural isolates (Table 3; Fig. 2C). The top-performing natural isolates (AXL64653.1, QOW77866.1, UNY47950.1, and UNY47954.1) originated from avian *Dependoparvoviruses*. Interestingly, AXL64653.1, UNY47950.1, and UNY47954.1 share high sequence similarity, differing by only a few point mutations, and were all isolated from chickens. The variant QOW77866.1 was isolated from a goose *Dependoparvovirus* and is not as closely related to the prior three. Of the engineered disulfide-containing variants, we observed certain combinations more frequently than others (Table 3). Specifically, disulfide C3 was present in the top-performing isolates C1/3, C2/3, and C1/2/3. The disulfide C4 seemed not to be significantly enriched by itself or with another disulfide, which implies that C4 is uniquely detrimental to viral propagation. This generally implies that wild-type virions can harbor disulfide bonds within certain positions of the PLA_2_ domain and remain propagating clones, in contrast to the widely accepted notion that PLA_2_ domains must transit the fivefold pore in a linear manner. In subsequent sections, the highly enriched disulfide variants C1/3, C2/3, and C1/2/3, along with the natural isolates AXL64653.1, QOW77866.1, UNY47950.1, and UNY47954.1, are evaluated for translatability to a recombinant vector setting. Because disulfide C3 was not enriched as a top isolate, but the disulfide was shared with C1/3, C2/3, and C1/2/3, we also chose to generate C3 as well.

**TABLE 3:** Performance of select top-performing and control variants^*a*^

Class	Variant	% P0 reads	% P1 reads	% P2 reads	Fold change P1	Fold change P2
Disulfide	C1/3	0.072	0.215	1.340	2.97	18.5
Disulfide	C2/3	0.113	0.456	1.306	4.04	11.5
Disulfide	C1/2/3	0.140	0.266	1.191	1.9	8.5
Avian dependoparvovirus	UNY47950.1	0.066	0.013	0.646	0.19	9.7
Avian dependoparvovirus	UNY47954.1	0.015	0.127	0.782	8.38	51.7
Avian dependoparvovirus	AXL64653.1	0.057	0.051	0.769	0.88	13.4
Avian dependoparvovirus	QOW77866.1	0.106	0.392	0.871	3.69	8.2
Human dependoparvovirus	AAV9	0.059	0.051	0.102	0.86	1.7
Calcium loop variant	D69A	0.008	ND	ND	ND	ND
Calcium loop variant	D69N	0.070	0.152	0.265	2.16	3.78
Calcium loop variant	D76A	0.052	0.038	0.027	0.73	0.52
Calcium loop variant	D76N	0.055	0.076	0.061	1.39	1.12
Active site variant	H75A	0.053	0.013	ND	0.24	ND
Active site variant	D97A	0.042	0.063	ND	1.50	ND
Active site variant	D97N	0.046	ND	0.014	ND	0.29

^*a*^ Both negative control and lead candidate PLA_2_ domains from the *in vitro* cycling performance in terms of library representation and fold change through directed evolution. ND, not detected.

By comparison, parental AAV9 showed modest enrichment, while attenuating mutations—such as those encoding active site ablations and calcium-binding loop variants—exhibited reduced or small enrichment, serving as controls (Table 3). These results recapitulate prior work on the AAV2 PLA_2_ domain, demonstrating that the equivalents of D69A, H75A, and D97A are lethal to AAV9 (16, 30). Interestingly, the mild enrichment of D69N and D76N individual capsid variants was unexpected, as neither had been previously evaluated in isolation. One possible explanation is that, compared to alanine substitutions, which significantly underperformed, asparagine replacements may confer partial functionality, likely due to the structural similarity of asparagine to aspartic acid. However, their low overall enrichment suggests that they do not enhance AAV9 transduction. These findings suggest that while the PLA_2_ calcium-binding loop exhibits some tolerance to mild substitutions, the predicted active site dyad remains highly constrained. These results suggest that iterative selection effectively filtered out non-functional PLA_2_ domains while enriching for variants capable of supporting AAV9 transduction.

### PLA_2_ domain compatibility is determined by the interaction between viral genera and the host

In all tested groups, the distribution between best- and worst-performing variants generally increased over successive infectious cycles. However, the predominant trend was depletion of non-viable PLA_2_ sequences, reinforcing that most of the explored sequence space is not functionally compatible with AAV9. Markov matrix generation of PLA_2_ was not productive at generating high-performing clones; however, the group’s mean remained consistent through evolution (Fig. 2D). Disulfide engrafted AAV9, which were designed to increase rigidity and possibly enzymatic activity, generally outperformed, presumably retaining functionality from the parental AAV9 (Fig. 2D). Statistically significant underperformance was observed in several genera, including *Protoparvoviruses*, *Copiparvoviruses*, *Tetraparvoviruses*, ambiguous *Parvoviridae*, and unknown/other (where the annotation was as group XIII, but the genera was non-parvoviral or not given in the NCBI database). Notably, several groups seemed to have neutral median fold change, indicative of a sequence space that comprises sequences both enhancing and limiting to AAV infection, for example, *Dependoparvoviruses*, *Bocaparvoviruses*, and *Erythroparvoviruses*. These observations suggest that functional constraints limit cross-genera interchangeability (Fig. 2E). When variants were analyzed based on their host species of origin, PLA_2_ sequences from porcine, non-porcine ungulate, and insect parvoviruses were significantly depleted (Fig. 2F).

**Fig 3:**
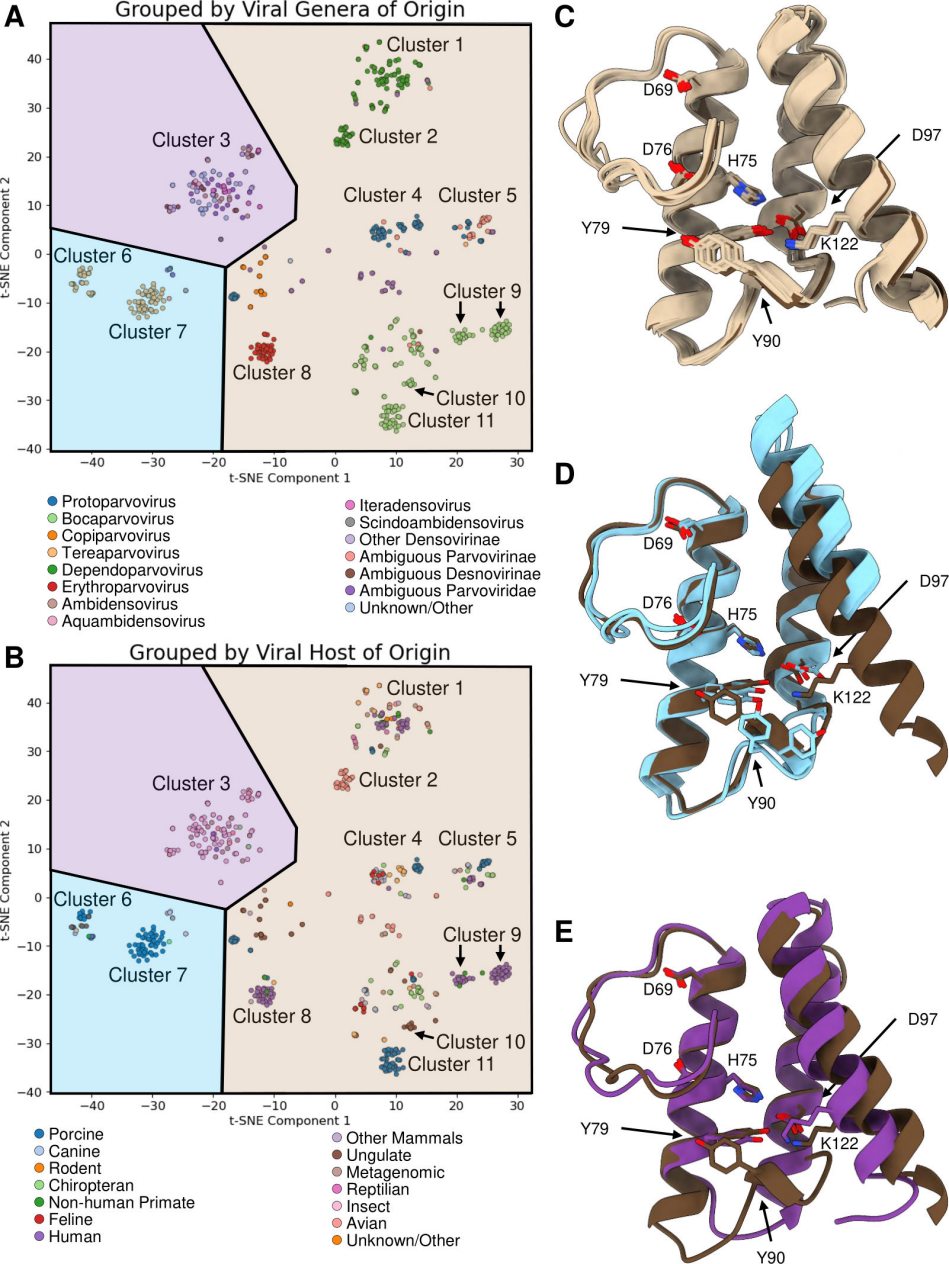
Embedding and clustering of natural parvoviral PLA_2_ variants reveal host-genera interactions. ESM2 embeddings were plotted in t-SNE space by components 1 and 2 and classified in a supervised manner by the (**A**) genera of origin or (**B**) host of isolation. Clusters of interest 1–11 are highlighted in the t-SNE plot. (**C**) AlphaFold3 structure of the swapped domain for a prototype specimen of Clusters 1, 2, 4, 5, 8, 9, 10, and 11 (tan), and AAV9 (brown). (**D**) AlphaFold3 structure of the swapped domain for a prototype specimen of 6 and 7 (pale blue), and AAV9 (brown). (**E**) AlphaFold3 structure of the swapped domain for a prototype specimen of 3 (purple) and AAV9 (brown). (**C–E**) The residues or residue equivalents to AAV9 D69, H75, D76, Y79, Y90, D97, and K122 are shown if conserved.

We hypothesize that host origin and genera likely interact to form valid PLA_2_ sequence space. To better decipher the host-genera interacting space, sequence embedding and t-SNE were used to classify all variants (Fig. 3A and B). Porcine isolates belong to the genus *Tetraparvovirus* (clusters 6 and 7), with some belonging to *Bocaparvovirus* (cluster 11), and to a lesser extent, to *Dependoparvovirus* and *Protoparvovirus*. The non-porcine ungulate viruses also belong to the genera *Tetraparvovirus* (cluster 6) along with *Bocaparvovirus* (cluster 10) and *Copiparvovirus*. Both porcine and non-porcine ungulates exhibited a general reduction in fold change in our screen, as did the genera *Tetraparvovirus* and *Copiparvovirus,* demonstrating the covariance of these observations. While individual densoviral genera were not statistically worse performing, sequences from insect isolates were significantly depleted. This could be due to the under-representation of sequences in these specific genera, leading to reduced statistical power when analyzed by that metric. However, the individual genera of *Densoviridae* are highly similar in sequence space, as indicated by their general association with cluster 3. Notably, *Dependoparvovirus* forms two distinct clusters. Cluster 1 is representative of broad species origin and is where human isolates (including AAV9) are found, while cluster 2 is a satellite exclusively of avian origin. The other clusters of note are cluster 4: *Protoparvoviruses* of feline/canine origin; cluster 5: *Protoparvoviruses* of non-canine/feline origin; cluster 8: *Erythroparvoviruses* of human origin; and cluster 9: *Bocaparvoviruses* of human origin. Overall, these results indicate that PLA_2_ sequence space is shaped by host and genus constraints, possibly limiting domain interchangeability.

The sequence embeddings reflect the fold topology, with clusters having unique and shared structural features. All folds share the common calcium loop binding residues D69 and D76, along with active site residue H75, which were defined in the initial bioinformatic search along with Y79. Clusters 1, 2, 4, 5, 8, 9, and 11 are all very similar with no insertions or deletions from AAV9 (Fig. 3C). Clusters 6 and 7 share a common fold consisting of a +10 amino acid insertion between αB and αC, a large enough insertion that it appears the C-terminus of the domains is largely truncated (Fig. 3D). This large truncation is likely a large influence on the poor performance of the *Tetraparvovirus* in our screen. Finally, cluster 3 has a small −5 amino acid deletion of αA and the AB loop, along with a +4 insertion extending the C-terminus of αB (Fig. 3E).

Of note, the top-performing natural isolates AXL64653.1, UNY47950.1, and UNY47954.1 all belong to cluster 1, while the variant QOW77866.1 belongs to cluster 2. Most of the other avian isolates in the library belonged to cluster 2. This implies that avian-originating parvoviruses are a potential reservoir of human viruses, in terms of this specific domain. This pattern suggests that avian-derived PLA_2_ sequences may occupy an evolutionary niche that allows for functional substitution in AAV9, potentially due to similarities in intracellular trafficking or capsid-host interactions. These observations may also imply that bovine-originating isolates are uniquely incompatible with AAV infection, and that the likelihood of human-cattle parvoviral chimeras is less than those with avian isolates.

### Enrichment of PLA_2_ domain swaps is not determined by improved viral productivity

To determine whether differences in viral yield during production contributed to the observed enrichment of the lead PLA_2_ candidates, we evaluated key viral components in a recombinant setting, including the relative abundance of P40Major and P40Minor mRNA transcripts, VP protein incorporation, vector yields, and genome packaging.

**Fig 4:**
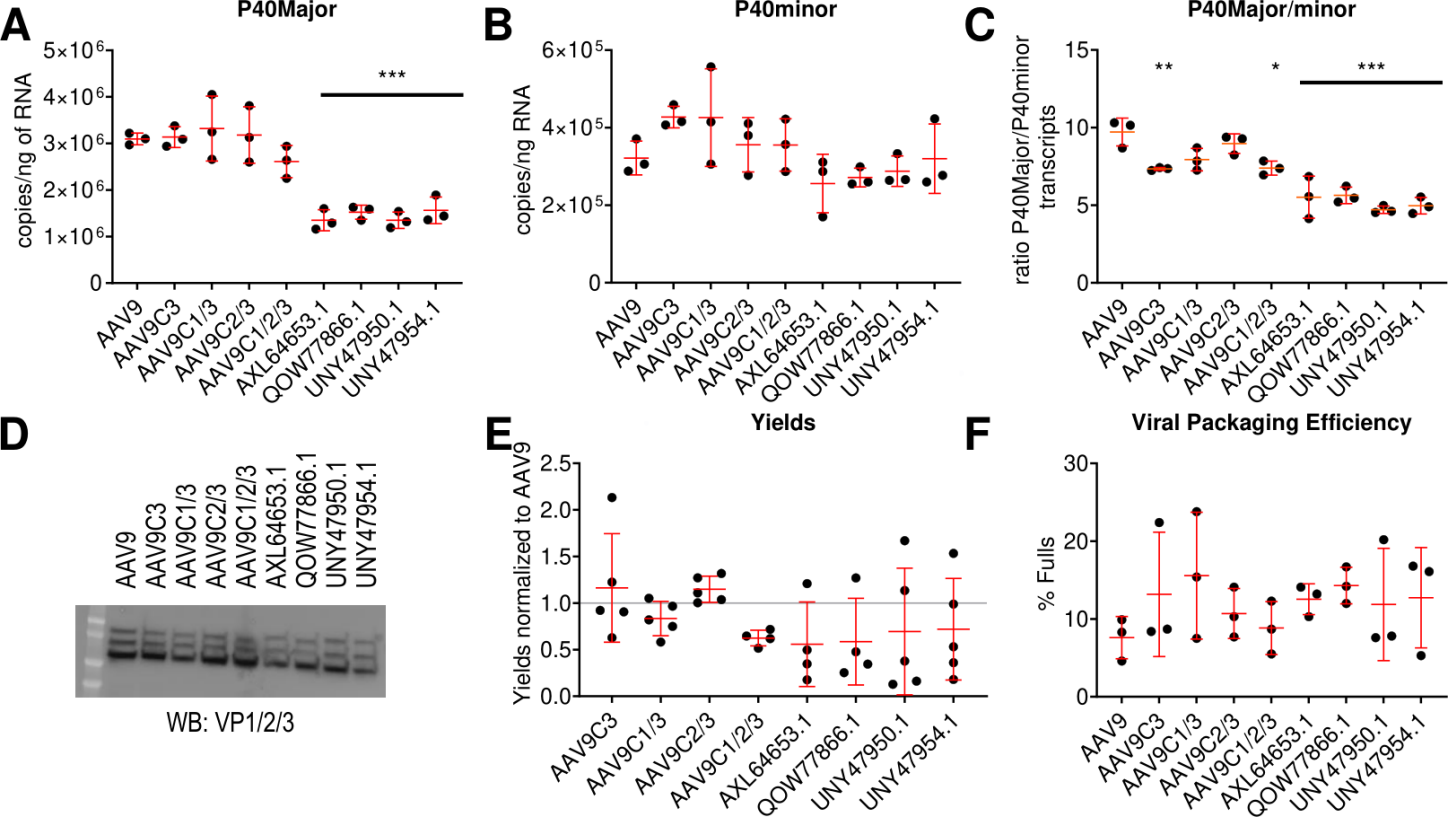
Chimeric AAV9/PLA_2_ vectors demonstrate altered mRNA splicing but retain capsid composition and packaging. Three days post-transfection, (**A**) P40 Major and (**B**) P40 minor transcript abundance was determined. (**C**) The ratio of the P40Major/P40Minor transcripts 3 days post-transfection was determined relative to the prior data. (**D**) Transiently transfected cells were lysed with RIPA buffer and run on a western blot of the capsid VPs in equal volume to determine the relative abundance of VP expression in the cells. (**E**) Vector genomes produced in suspension culture relative to AAV9 produced in the same batch. (**F**) Determination of the percentage of full particles for different preparations. One-way ANOVA: *P < 0.05; **P < 0.01; and ***P < 0.001.

Natural isolates exhibited a significant reduction in P40Major transcript levels compared to AAV9, while P40Minor transcripts remained largely unchanged (Fig. 4A and B). This resulted in an overall decrease in the P40Major/P40Minor ratio (Fig. 4C), suggesting that these variants may produce virions with altered VP composition. However, despite this shift in mRNA expression, total VP protein levels determined by western blot were reduced across all capsid proteins in natural isolates, with no evidence of a VP1/VP2 ratio increase that would typically indicate enhanced VP1 incorporation (Fig. 4D).

To further assess whether production efficiency contributed to the observed selection, we quantified total vector genome yields. Natural isolates exhibited a modest reduction in vector production, but the difference was not statistically significant (Fig. 4E). This strongly implies that these variants were not selected for enhanced productivity at a genomic level. Additionally, mass photometry was performed to assess the proportion of full vs empty virions. No significant differences in the percentage of full virions were observed between natural isolates, AAV9, or disulfide-engineered variants (Fig. 4F). This again suggests that these variants exhibit no unique advantage in genome packaging that would cause their enrichment.

Taken together, these findings suggest that viral progeny generation, VP expression, genomic yields, and genomic packaging are all unlikely to be the cause of enrichment of our lead candidates. Instead, we hypothesized that post-entry mechanisms, such as intracellular trafficking and nuclear import, may be responsible for their enrichment during infectious library cycling.

### Recombinant chimeric virions carrying highly enriched PLA_2_ domains display improved transduction

**Fig 5:**
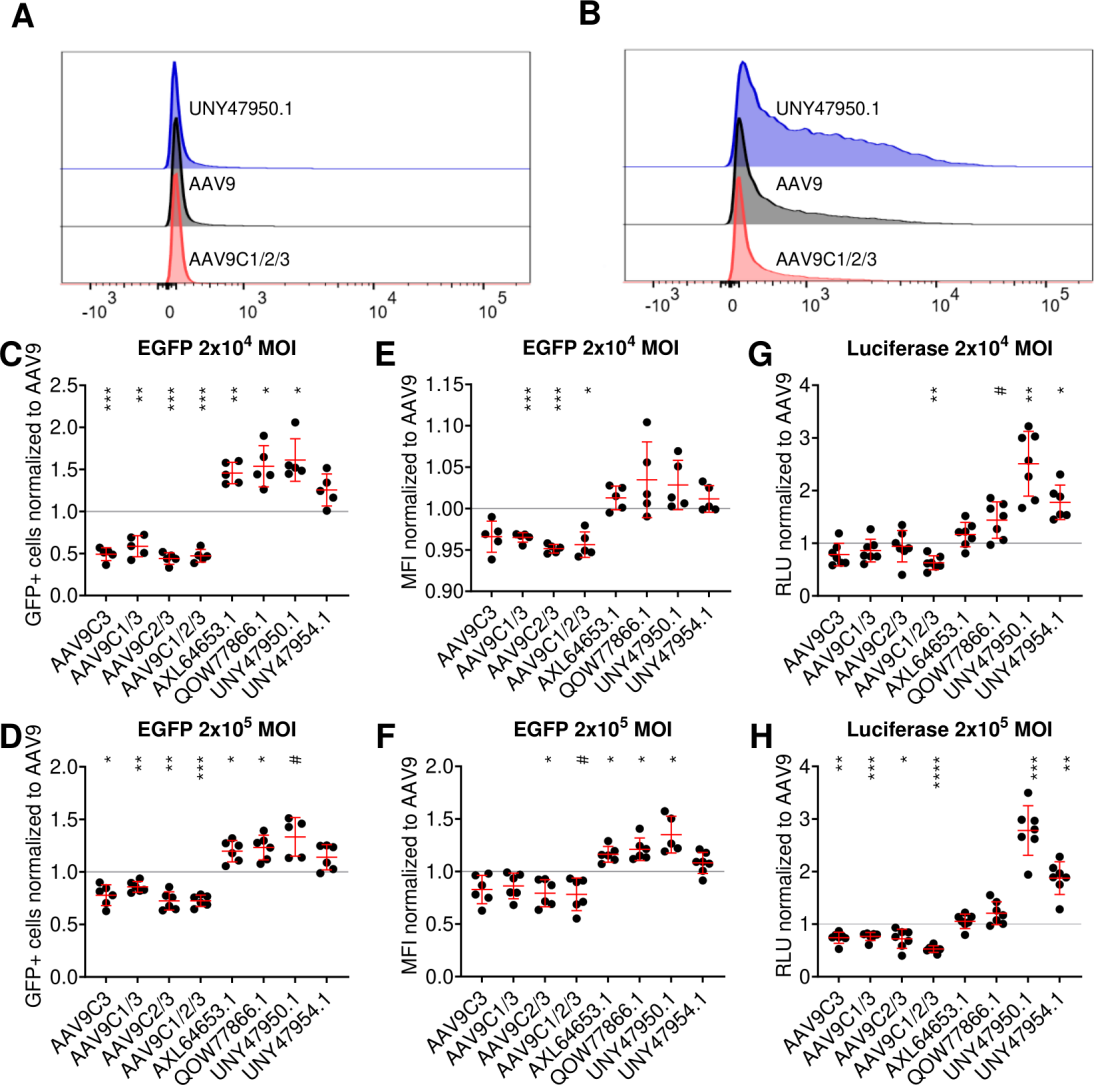
Chimeric AAV9/PLA_2_ vectors packaging single-stranded or self-complementary genomes demonstrate improved transduction efficiency. Self-complementary EGFP vectors transduced cells at (**A**) low and (**B**) high MOI and quantitated by flow cytometry. Representative histograms for AAV9, AAV9C1/2/3, and UNY47950.1 are shown. The percentage of EGFP+ cells at (**C**) low and (**D**) high MOI. Mean fluorescent intensity of EGFP+ cells at (**E**) low and (**F**) high MOI. Luciferase expression at (**G**) low and (**H**) high MOI. One-way ANOVA: #*P* < 0.1; *P < 0.05; **P < 0.01; ***P < 0.001; and ****P < 0.0001.

To assess whether the increased replication of AAV capsids carrying non-mammalian PLA_2_ candidates translates into a recombinant vector setting, we evaluated the transduction efficiency of both self-complementary EGFP and single-stranded luciferase reporter-based vectors at low and high multiplicities of infection. Under all conditions tested, AAV9/PLA_2_ domain chimeras containing disulfide bonds exhibited reduced transduction efficiency in the context of scEGFP vectors as reflected by both the percentage of EGFP+ cells and mean fluorescence intensity. These results suggest that while disulfide bridges may stabilize parvoviral PLA_2_ domains, they appear to impair function, possibly by disrupting the flexibility and structural dynamics required for efficient extrusion from the capsid interior and enabling subsequent infectious steps required for efficient transduction. These results further corroborate the importance of PLA_2_ function (and hence infection) over viral titer in determining selective pressure. In contrast, natural PLA_2_ isolates either matched or slightly outperformed AAV9, indicating that they retained or improved PLA_2_ functional activity (Fig. 5A through F). To further validate these findings, we performed luciferase assays using single-stranded luciferase vectors at low and high MOI. The results mirrored those of the EGFP assay, with disulfide-containing AAV9 PLA_2_ variants underperforming across all conditions and natural isolates either matching or exceeding AAV9 transduction efficiency (Fig. 5G and H). Among the natural PLA_2_ variants, UNY47950.1 exhibited the highest transduction efficiency across both vector formats, making it the top-performing candidate. Based on these findings, UNY47950.1 was selected for further mechanistic studies to assess its impact on intracellular trafficking and nuclear entry.

### UNY47950.1/AAV9 chimeric virions show improved post-entry trafficking

**Fig 6:**
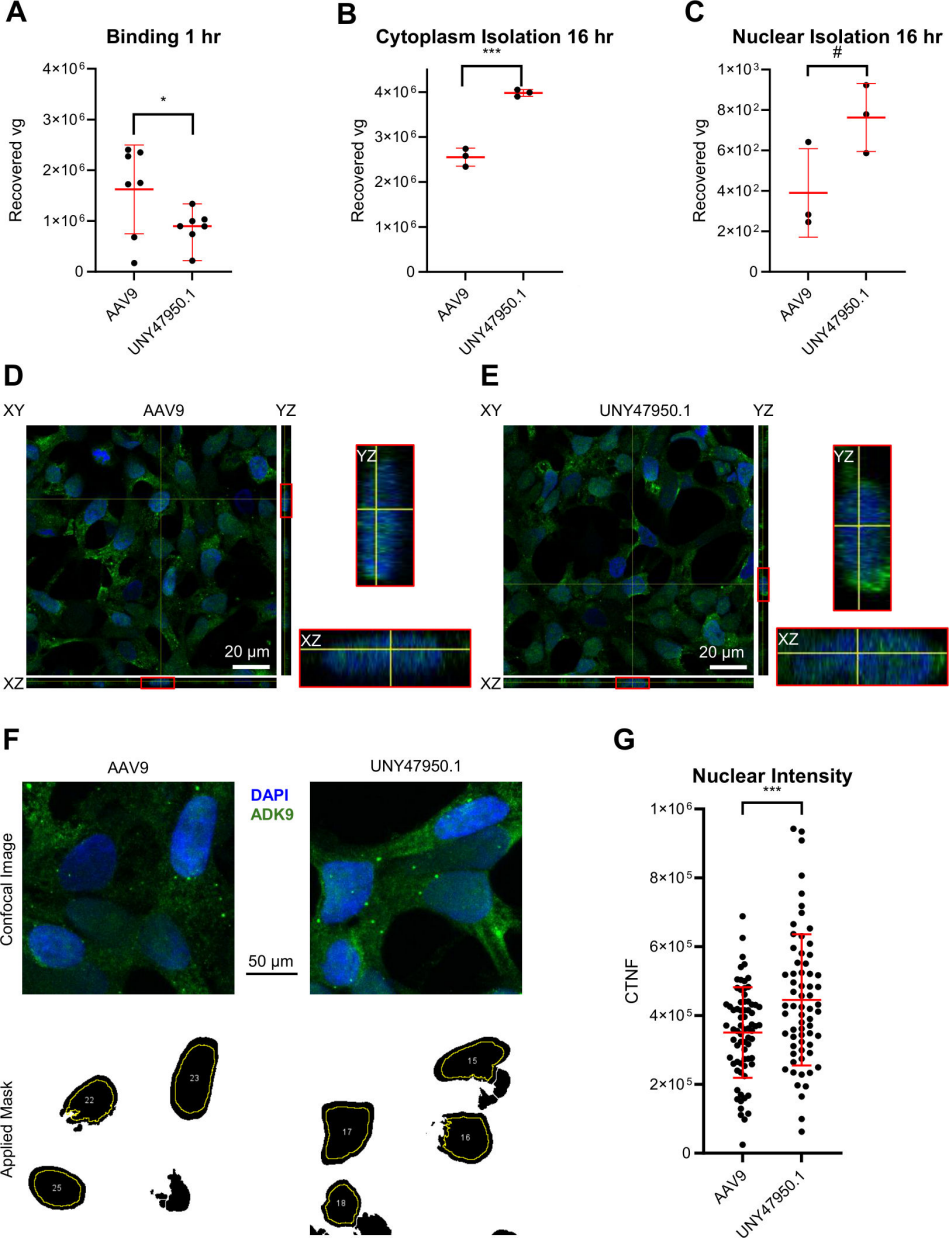
Cell surface binding and cytoplasmic and nuclear localization of chimeric AAV9/PLA_2_ vectors. Quantitative PCR-based assays were conducted to detect vector genome (**A**) binding after 1 h at 4°C, (**B**) in the cytoplasm after 16 h at 37°C, and (**C**) in the nucleus after 16 h at 37°C. (**D**) Confocal microscopy after 16 h at 37°C of AAV9. Shown in the XY plane is a representative plane from the center of the stack. The XZ and YZ planes are shown adjacent. Inset to the right is the XZ and YZ of a single cell of focus, demonstrating the nuclear signal of AAV9. (**E**) Confocal microscopy after 16 h at 37°C of AAV9/UNY47950.1. Shown in the XY plane is a representative plane from the center of the stack. The XZ and YZ planes are shown adjacent. Inset to the right is the XZ and YZ of a single cell of focus, demonstrating the nuclear signal of AAV9/UNY47950.1. (**F**) Representative maximum projections of the prior panels of AAV9 (left) and AAV9/UNY47950.1 (right) staining for capsids and nuclei (top) and with the mask used to quantify the fluorescence intensity within the nuclei (bottom, black is DAPI signal and yellow line is the mask where capsid signal was quantitated). (**G**) Quantification of fluorescence intensity within the nuclei of cells. Student’s *t*-test: #*P* < 0.1; *P < 0.05; and ***P < 0.001.

To gain further insight into the infectious biology of UNY47950.1/AAV9, we first assessed cellular binding and uptake of these virions. Since PLA_2_ modifications occur within the VP1u region and not on the capsid surface, one would expect cell binding to remain unchanged. However, we find chimeric UNY47950.1/AAV9 to have slightly impaired cell binding (Fig. 6A). However, when intracellular trafficking was assessed at 16 h post-infection, the chimera demonstrated improved intracellular trafficking, with higher vector genome levels detected in both the cytoplasm (Fig. 6B) and nucleus (Fig. 6C), suggesting enhanced post-entry activity.

To further validate these findings, confocal microscopy was used to visualize vector localization. At 16 h post-infection, the chimeric AAV capsid exhibited stronger nuclear accumulation than AAV9 as indicated by staining for intact viral capsid protein with antibody ADK9 (Fig. 6D and E). We further quantified this nuclear fluorescence intensity by masking the DAPI-stained nuclei (Fig. 6F) to enable per-cell quantification (Fig. 6G). These results indicate that the UNY47950.1 PLA_2_ domain enhances AAV infection at a post-entry stage, likely by improving cytoplasmic trafficking and nuclear import.

To assess whether this functional orthogonality extends beyond AAV9, we tested a sero-distinct AAV5 chimera carrying the UNY47950.1 PLA_2_ domain. The UNY47950.1/AAV5 chimera also retained infectivity, while exhibiting a modest improvement in transduction efficiency compared to parental AAV5 (117% ± 10% of AAV5, *n* = 3, *P* < 0.05). This suggests that UNY47950.1 alteration to post-entry trafficking may not be strictly dependent on AAV capsid surface-mediated interactions with host cells but may represent a more broadly compatible mechanism across multiple AAV serotypes.

## DISCUSSION

In this study, the functional orthogonality of parvoviral PLA_2_ domains is probed in the context of an AAV capsid. By exploring a large panel of natural isolates, synthetic, and variant PLA_2_ domains, key insights into parvoviral infectivity have emerged with regard to the structure and function of this essential domain.

In general, we find that closely related sequences are more orthogonal than distant relatives. Notably, three of the isolates that enabled increased replication of wtAAV (AXL64653.1, UNY47950.1, and UNY47954.1) are point variants of each other, highlighting that distinct optima exist in the evolutionary landscape of this domain for parvoviruses. In contrast, the engineered PLA_2_ variants containing disulfides, while enriched during selection, underperformed in the context of recombinant vectors. It has previously been hypothesized that the group XIII PLA_2_ of parvoviruses likely selected against disulfides to allow VP1u domain externalization via the AAV capsid fivefold pore. One can speculate that such an event would require unfolding and linear transit of the domain through the pore, which would not be structurally compatible with disulfide bridges. The moderately reduced infectivity suggests that excess rigidity in the structure does indeed hamper transduction. However, the fact that there is any viability of these candidates demonstrates that disulfides may be reversible and amenable to unfolding, or that the PLA_2_ domain may be able to escape in a molten globule for externalization. However, this may require capsid changes of a larger scale than previously anticipated by the fivefold hypothesis and could lend further evidence toward the fivefold iris (wherein the fivefold must expand for PLA_2_ trafficking) or twofold externalization (wherein the twofold must rearrange for externalization) hypothesis (29).

The observed depletion of distantly related PLA_2_ genera, along with the maintenance of relatively few genera, emphasizes the evolutionary constraint on PLA_2_ functionality. For *Erythroparvovirus* B19, it has been demonstrated that a region N-terminal to the PLA_2_ domain is essential for its optimal enzymatic function, but not for folding or minimal (~10%) activity (19). Additionally, flanking regions of the core PLA_2_ domain are essential in B19 for productive infectious function (24). However, even *Erythroparvovirus* sequences, such as B19, were relatively maintained over two viral passages without this region, demonstrating this genus to be generally more compatible than many others. Furthermore, in addition to D69, H75, and D76, several residues appear to be conserved from a structural position standpoint but not just based on sequences we queried in our literature search. These include Y79 (23) and Y90 (with the exception of cluster 3), and the putative dyad aspartic acid D97. Additionally, K122 was identified in all clusters except for clusters 6 and 7, where it was deleted in the swap, a result of those domains having insertion of the BC hinge relative to AAV9. Inspection of the source sequences from clusters 6 and 7 reveals a lysine +4 from the last swapped residue (e.g., AZZ69400.1 and AGM20585.1), which would occupy the same position. The conservation and positioning of Y79 and K122 within the active site imply that they are possibly a part of the catalytic mechanism or water network for active site regeneration. These findings indicate that structural features beyond the PLA_2_ core—such as domain extensions and host/capsid interactions—may impose additional constraints on functional interchangeability.

It is important to note that the present study focused on swapping the PLA_2_ domain within the constraints of available oligo pool synthesis methods at the time of library construction. Future studies expanding the boundaries of domain interchangeability—by incorporating extended N- and C-terminal regions—may uncover additional viable sequence space (notably within clusters 6 and 7 PLA_2_). Nevertheless, our findings strongly suggest that *Dependoparvovirus* sequences of avian origin represent a functionally compatible and evolutionarily viable reservoir for PLA_2_ domain replacement in primate-derived AAVs.

Despite strong enrichment during infectious cycling, natural PLA_2_ isolates exhibited lower VP protein expression and altered P40Major/P40Minor splicing relative to AAV9. However, these differences did not affect the proportion of full capsids or VP1 incorporation. This suggests that the swapped PLA_2_ region influences not only splicing regulation but also overall VP protein expression. The lack of altered VP ratios demonstrates that there is likely a maximum quantity of VP1 that may be incorporated into capsids before capsid assembly is disrupted. This is supported by the prior literature, as VP1 and VP2 are unable to assemble capsids despite their shared C-terminal core with VP3 (44). The natural variants, when evaluated as recombinant vectors, have moderately improved transduction. Enhanced cytoplasmic and nuclear localization determined by genome detection and confocal microscopy implies that these capsids may bypass cellular barrier(s) or exploit cellular machinery more efficiently than AAV9. Notably, escape from the endo-lysosomal or trans-Golgi apparatus prior to nuclear entry is presumed to be facilitated by the PLA_2_ domain (16, 19, 21–23). However, others have postulated a role in nuclear envelope breakdown for AAV import into the nucleus, another step that may be impacted by PLA_2_ domain swaps (45). Hence, with regard to UNY47950.1, it is quite possible that the PLA_2_ domain is responsible for improved cytoplasmic and/or nuclear entry. Another attribute of VP1u domains described recently is their ability to alter epigenome expression, which has been previously described (15, 46). While this is certainly plausible in the case of some of the domains in our capsid library, at least in the case of UNY47950.1, the vector genome copies recovered from the cytoplasm and nuclei are accompanied by increasing transgene expression, implying that epigenome modifications are likely not at play for that variant.

Interestingly, UNY47950.1 was not the best-performing variant in selection, ranking as the fifth best. In addition, the degree of enrichment was not indicative of the magnitude of performance during recombinant vector transduction. Several factors could contribute to these discrepancies. First, when produced by the transfection method, AAV cross-packaging is a known issue that only becomes more pronounced as library size decreases (43). Second, because producer cells are used for *in vitro* cycling, secreted virus may lead to re-infection of the producer cells, creating additional, untracked selection cycles. Together, these factors suggest that the enrichment magnitude in NGS does not directly reflect transduction performance. Nevertheless, it is still indicative of a viable PLA_2_ sequence space compatible with AAV. Importantly, the ability of UNY47950.1 to function within both AAV9 and AAV5 capsids, which are structurally, serologically, and functionally divergent, further demonstrates that the core PLA_2_ domain may retain modularity across serotypes. Overall, these findings expand our understanding of PLA_2_ domain evolution, structure-function relationships, and cross-serotype compatibility. Interestingly, it has been described that the VP1u domain is particularly prone to generating T- and/or B-cell epitopes, and the ability to swap existing PLA2 domains with divergent alternatives may provide an opportunity to generate capsids that can evade such adaptive host processes (3, 13, 47). Overall, these biological insights may help optimize AAV entry and intracellular trafficking in the context of recombinant vectors, ultimately improving their potency and efficiency for gene transfer applications.

## Data Availability

Supplemental Data Set S1 lists accession numbers and sequence information of different parvoviral PLA2 domains, as well as next-generation sequencing data for different viral passages, including raw read counts and *P* values.

## SUPPLEMENTAL MATERIAL

Data Set S1 jvi.00799-25-s0001.xlsx Accession numbers, sequence information, and next-generation sequencing data.

Fig. S1 jvi.00799-25-s0002.docx EGFP flow cytometry gating strategy.

ASM does not own the copyrights to Supplemental Material that may be linked to, or accessed through, an article. The authors have granted ASM a non-exclusive, world-wide license to publish the Supplemental Material files. Please contact the corresponding author directly for reuse.
