# Supplementary material for: Functional orthogonality of parvoviral phospholipase A2 domains in adeno-associated virus transduction
Source: J Virol. 2025 Aug 12;99(9):e00799-25. doi: 10.1128/jvi.00799-25 (PMC12455919; doi:10.1128/jvi.00799-25)
Supplement: Fig. S1 — EGFP flow cytometry gating strategy. [file jvi.00799-25-s0002.docx]

**Figure S1. EGFP flow-cytometry gating strategy.**  Gating strategy is show for a representative experiment for selected conditions in the rows of cells only control, AAV9, AAV9C1/2/3, and UNY47950.1. Discrimination of cells, doublet/singlet, and gating are shown in columns. The percentages in the graphs are the percent of the parent population which are positive within a given gating parameter.
